# Selected elements of the lifestyle of Silesian seniors, taking into account their participation in the activities of the Third Age Universities

**DOI:** 10.3389/fpubh.2024.1375238

**Published:** 2024-03-04

**Authors:** Józefa Dąbek, Magdalena Szynal, Ewelina Łebek, Oskar Sierka

**Affiliations:** ^1^Department of Cardiology, Faculty of Health Sciences in Katowice, Medical University of Silesia, Katowice, Poland; ^2^The Doctoral School of Medical University of Silesia in Katowice, Medical University of Silesia, Katowice, Poland

**Keywords:** seniors, Universities of Third Age, physical activity, stress, depression

## Abstract

**Introduction:**

UTA can provide older adult people with the satisfaction of needs and creates the opportunity to pursue youthful interests and passions. The aim of the study was to assess selected elements of the lifestyle of Silesian seniors, taking into account their participation in the activities of Universities of the Third Age.

**Methods:**

The study involved 631 (100%) senior residents of the Silesian agglomeration. The majority of the study group were women (475; 75.28%), and the average age of the participants was 70.28 ± 6.09 years. To conduct the study, an original survey questionnaire was used, complemented by PPS-10, PAQE and Yesavage Geriatric Depression Rating Scale.

**Results:**

Among the surveyed Silesian seniors who did not attend classes at the University of the Third Age, a statistically significantly higher score on the Yesavage’s Geriatric Depression Rating Scale was found compared to those confirming their participation in the mentioned activity (*p* = 0.002). Almost 40% (107; 38.63%) of seniors who did not attend classes at the Universities of the Third Age showed a high level of stress, and every fourth (89; 25.14%) Silesian senior taking part in the above-mentioned activity had a low level of stress (*p* = 0.04). The median of points obtained on the physical activity assessment scale (PAQE) by seniors attending classes at Universities of the Third Age was statistically higher than seniors who denied participation in the mentioned activity (*p* = 0.017).

**Conclusion:**

Participation in the various activities at the Universities of the Third Age influenced positively well-being, reduced stress and raised physical activity of examined seniors. It is important to promote and start actions leading to seniors’ better and easier inclusion to the society life. Future research should concentrate on reasons why many seniors do not attend activities in their leisure time - especially on accessibility of various activities and financial reasons, which in the future will play crucial role in the aging societies.

## Introduction

Physical activity is one of the elements of a healthy lifestyle. According to the recommendations of the World Health Organization (WHO), healthy adults should undertake: moderate exercise (at the level of 3–6 MET for 150–300 min/week) or intense exercise (6 or more MET for 75–150 min/week) or an equivalent combination of moderate and intense exercise (1). The latest recommendations of the Institute of Food and Nutrition are also presented in the form of the “Pyramid of Healthy Eating and Physical Activity” at its base (2).

In Poland, the level of physical activity in society is very low, even disturbing. Moreover, with age, the number of people exercising regularly decreases. The study conducted by the Ministry of Sport and Tourism entitled “Level of physical activity of Poles 2018” shows that only 21.8% of Poles met WHO standards regarding the level of physical activity in free time (3).

Regular and systematic physical activity affects, among others: increasing the efficiency of the circulatory system, lowering blood pressure, increasing the stroke volume of the heart and improving the elasticity of blood vessels and reducing the risk of developing atherosclerosis and its complications. In addition, it reduces the risk of stroke, improves metabolism and, consequently, the treatment of obesity and overweight, reduces stress, improves cognitive functions and improves logical thinking processes, concentration of attention and memory. It also contributes to improving well-being (4–7). Researches proves, that regular physical activity promotes better quality and even extension of life (5, 7). The basic task of physical activity in older people is to maintain the appropriate level of psychophysical fitness and physical capacity for as long as possible.

In Poland, the first University of the Third Age (UTA) was established in 1975 in Warsaw as part of the Postgraduate Center for Medical Personnel Education, and was founded by prof. Halina Szwarc. Its aim was to enable older people who could not receive education in their youth to acquire knowledge, implement a continuing education program, conduct gerontological research and improve the quality of life of the older generation. Nowadays, UTA can provide older people with the satisfaction of needs such as: self-education, learning about the environment, being in a group, acceptance, expanding knowledge and skills, filling free time, mental and physical stimulation, learning new technologies and ways of communicating. Moreover, UTA creates the opportunity to pursue youthful interests and passions. Attending classes organized within the UTA allows seniors to undertake various forms of physical activity, shape a healthy lifestyle, but also raise awareness of the importance of exercise in everyday life (8). The aim of the study was to assess selected elements of the lifestyle of Silesian seniors, taking into account their participation in the activities of Universities of the Third Age.

## Materials and methods

The research began after obtaining the consent of the Bioethics Committee of the Medical University of Silesia in Katowice - resolution no. PCN/0022/KB1/36/21. The study involved 631 (100%) senior residents of the Silesian agglomeration. The majority of the study group were women (475; 75.28%), and the average age of the participants was 70.28 ± 6.09 years. All methods used in this study were in accordance with applicable guidelines and regulations for conducting scientific research (9).

Inclusion criteria for the study included: age (≥60 years), voluntary, informed consent to participate in the study, ability to follow instructions, ability to read, and no need for assistance from third parties/others in completing the questionnaire. All of the inclusion criteria listed were intended to eliminate any interference from third parties in the process of completing the survey.

For the purposes of statistical analyses, seniors were divided according to: participation in activities at Universities of the Third Age (Yes/No), gender (women/men), age (from 60 to 70 years, from 71 to 80 years), ≥ 81 years of age and education (primary, vocational, secondary, higher) and place of residence (urban/rural).

To conduct the study, an original survey questionnaire was used, containing questions regarding the issues included in the purpose of the work and basic questions including age, gender, place of residence, marital status and level of education.

The Polish version of the PPS-10 questionnaire was used to assess the severity of stress. The questions include an assessment of the intensity of stress related to one’s own life situation during the last month (10). Respondents rated the frequency of their feelings and thoughts about life events and situations using a five-point scale, where 0 means “never” and 4 means “very often.” The results of the PSS-10 test are expressed in points that reflect the level of stress experienced. Overall, the result can be interpreted as follows: 0–13 points: low stress. Indicates good control over stressors and the ability to cope with them, 14–26 points: average stress level. It may indicate a need to better manage stressors or seek ways to cope with stress, and 27–40 points: high stress level. It indicates a significant burden of stress and the need to take action to reduce it. The Polish version of the questionnaire used in the study was PSS-10 validated by Z. Juczyński and N. Ogińska-Bulik (11).

In order to assess the well-being of the subjects, the Yesavage Geriatric Depression Rating Scale was used. The mentioned scale consists of 15 statements to which each respondent was asked to respond by selecting “yes” or “no.” Appropriate calculations and summing up the points allow for the diagnosis of severe or moderate depression or its absence (12).

To assess the physical activity of seniors, the Polish version of the standardized PAQE questionnaire (The Physical Activity Questionnaire for the Elderly) prepared by Król-Zielińska M. et al. was used. This questionnaire provides quantitative data on usual physical activity (including home, sports and recreational activities) over the past year. The PAQE questionnaire is also known as the “modified Baecke questionnaire.” In described questionnaire, respondents were asked about their habitual physical activity during the last year, taking into account household, sport and other activities especially in leisure time. For household activity questions there are five possible answers ranging from very active (4 points) to inactive (0 points). The household score, calculated form 10 items, is the sum of all obtained points divided by 10. With regard to sports and other activities, information about the activity type, hours per week, and period of the year in which the activity is normally performed was obtained. The intensity codes (based on energetic costs of activities) were used to characterize the type of activities. Additionally, codes are provided for hours of the week and periods of the year. The sport and other leisure activity scores (points) are the result of the multiplication of the three codes mentioned above. The total result (points) is the sum of all activity domains (13).

Completing the survey and individual questionnaires was completely anonymous and voluntary. The methods used to collect the questionnaires (placing them in white unmarked envelopes after completing them, collecting the envelopes in one secured place, opening envelopes with completed questionnaires only when entering the obtained results into the database) made it impossible to identify the people participating in the study.

Statistica 13.3 (StatSoft Poland) was used to perform statistical analyses. Because the data obtained showed a non-normal distribution the study used non-parametric tests, including the Mann–Whitney U test and Kruskall-Wallis ANOVA. The Chi^2^ test was used for qualitative data. In all analyzes performed, the level of statistical significance was set at *p* < 0.05.

## Results

General characteristics of the study group are presented in the Table 1.

**Table 1:** General characteristics of the study group.

Studied group of seniors (*n* = 631; 100%)						
Variables					*n*	%
Sex				Women	475	75.28
				Men	156	24.72
Age [years]				60–70	373	59.11
				71–80	220	34.87
M	SD	Min.	Max.	≥81	39	6.18
70.28	6.09	60	96			
Level of education				Primary	37	5.86
				Vocational	120	19.02
				Secondary	292	46.28
				Higher	182	28.55
Place of residence				City	436	69.10
				Rural areas	195	31.90
Attending classes at the University of the Third Age				Yes	354	56.10
				No	277	43.90

n, number; M, average; SD, standard deviation; Min., minimum value; Max., maximum value.

The majority of the study group were women (475; 75.28%) and people aged 60 to 70 years (373; 59.11%). Most respondents had secondary education (292; 46.28%), and over 55% (354; 56.10%) of the surveyed group attended classes at Universities of the Third Age.

Table 2 presents the characteristics of the study group, including descriptive statistics and a results of differences analysis in the number of seniors with individual degrees of depression distinguished using the Yesavage’s Geriatric Depression Rating Scale Almost 40% (141; 39.83%) of seniors taking part in activities at the University of the Third Age did not show signs of depression, while severe depression was observed in 11 (3.97%) who denied participating in the above-mentioned activity. The observed differences in the number of seniors with particular degrees of depression severity between the compared groups turned out to be statistically significant (*p* = 0.004). This means that attending classes at Universities of the Third Age was associated with a lower risk of developing depression.

**Table 2:** Characteristics of the studied group of seniors, including the analysis of differences in the number of seniors with individual degrees of depression distinguished using the Yesavage’s Depression Assessment Questionnaire.

Seniors’ studied group (*n* = 631; 100%)							
Variables	UTA „+” (*n* = 354)		UTA „-” (*n* = 277)		Chi^2^		
	*n*	%	*n*	%	χ^2^	df	*p*
*n* i % of the whole group	354	56.10	277	43.90	11.27	2	0.004
*n* i % of a given group	354	100	277	100			
No depression	141	39.83	78	28.16			
Moderate depression	207	58.47	188	67.87			
Severe depression	6	1.69	11	3.97			

UTA “+”, seniors participating in classes at Universities of the Third Age; UTA “-”, seniors not participating in classes at Universities of the Third Age; n, number of participants; *χ*^2^, value of the Chi^2^ statistic; df, degrees of freedom, *p*, level of statistical significance.

**Figure 1:**
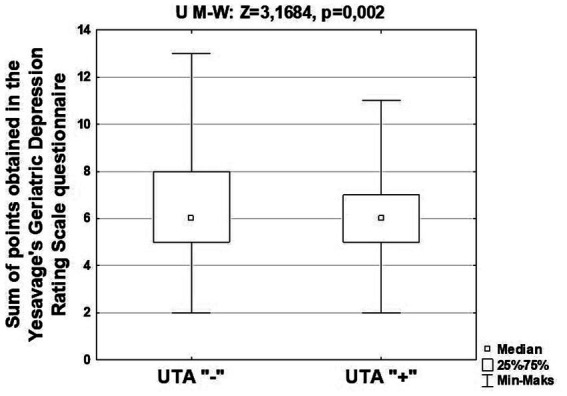
Characteristics of the studied group of Silesian seniors, taking into account the results of the analysis of differences in the total number of points obtained in the Yesavage’s Geriatric Depression Rating Scale.

Figure 1 shows the characteristics of the study group, taking into account the results of the analysis of differences in the total number of points obtained in the Yesavage’s depression assessment questionnaire by seniors attending classes at Universities of the Third Age and those denying participation in the mentioned classes. Supplementary Table S1 presents the characteristics of the studied group, taking into account the descriptive statistics of the points results obtained by seniors attending and not attending classes at Universities of the Third Age in the above-mentioned questionnaire, and Supplementary Table S2 presents the characteristics of the study group of seniors, taking into account the descriptive statistics of the points obtained in the Yesavage’s Geriatric Depression Rating Scale questionnaire and attending classes at Universities of the Third Age, sex, age, level of education and place of residence.

Among the surveyed Silesian seniors who did not attend classes at the University of the Third Age, a statistically significantly higher score on the Yesavage’s Geriatric Depression Rating Scale was found compared to those confirming their participation in the mentioned activity (*p* = 0.002). Based on the results of the above analysis, it can be assumed that participation in activities at Universities of the Third Age has a positive impact on well-being and reduces the risk of developing depression by maintaining social contacts.

The Kruskal-Wallis test showed statistically significant differences (*p* < 0.001) in the number of points scored between seniors aged 60 to 70 who attended (*p* < 0.01), aged 60 to 70 who did not attend (*p* = 0.008) and seniors aged 71 to 80 years old attending (*p* = 0.020) classes at universities of the third age compared to seniors aged 71 to 80 years old who denied participation in the activity in question, who obtained higher point values on the Yesavage’s geriatric depression rating scale than the others abovementioned seniors.

The analysis also showed a statistically significant difference (*H* = 29.545; *p* = 0.0001) between the number of points obtained in the Yesavage’s Geriatric Depression Rating Scale questionnaire between seniors with primary education (*p* = 0.01) and vocational education (*p* = 0.02) not participating in the activities of Universities of the Third Age, and seniors with higher education participating in the mentioned activity. Seniors with primary and vocational education obtained higher scores on the scale in question compared to respondents with higher education, which may indicate the protective influence of education on the possibility of developing depression among seniors.

The Kruskall-Wallis test also showed statistically significant (*H* = 23.655; *p* = 0.0006) differences in the number of points obtained by seniors living in the cities and taking part in the activities of the Universities of the Third Age, compared to those living in the countryside and denying participation in the discussed activity (*p* = 0.03). Therefore, it can be concluded that living in cities with good access to the discussed activities for seniors has an antidepressant effect. The results of the multiple comparisons described above are presented in the supplementary material (Supplementary Figures S1–S3; tables of multiple comparisons Supplementary Tables S3–S5).

**Figure 2:**
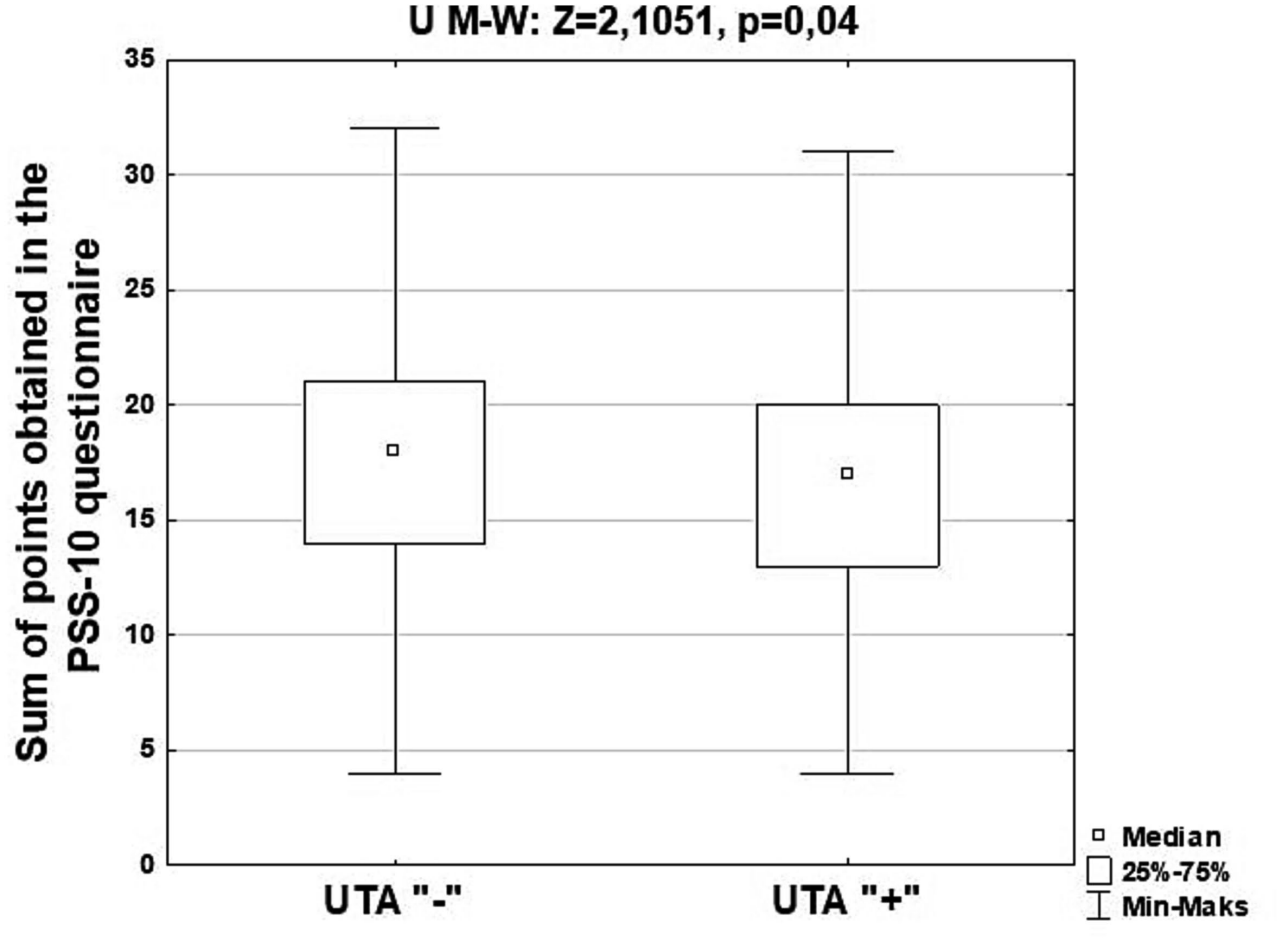
Characteristics of the studied group of Silesian seniors, taking into account the results of the analysis of differences in the total number of points obtained in the PSS-10 questionnaire by seniors participating in classes at Universities of the Third Age and denying the above-mentioned activity.

Table 3 presents the characteristics of the study group, taking into account the analysis of differences in the number of respondents with individual degrees of stress intensity distinguished using the PSS-10 stress severity assessment questionnaire, and Figure 2 - taking into account the results of the analysis of differences in the total number of points obtained in the PSS-10 questionnaire by Silesian seniors attending classes at Universities of the Third Age and denying the above-mentioned activity. Supplementary Tables S6, S7 present descriptive statistics of the studied group of Silesian seniors, taking into account the scores obtained in the PSS-10 stress assessment questionnaire and participation in classes at Universities of the Third Age, sex, age, level of education and place of residence.

**Table 3:** Characteristics of the study group, including the analysis of differences in the number of seniors with individual degrees of stress intensity distinguished using the PSS-10 stress severity assessment questionnaire.

Seniors’ studied group (*n* = 631; 100%)							
Variables	UTA „+” (*n* = 354)		UTA „-” (*n* = 277)		Chi^2^		
	*n*	%	*n*	%	*χ* ^2^	df	*p*
*n* i % of the whole group	354	56.10	277	43.90	5.94	2	0.04
*n* i % of a given group	354	100	277	100			
Low	89	25.14	52	18.77			
Moderate	157	44.35	118	42.60			
High	108	30.51	107	38.63			

UTA “+” - seniors participating in classes at Universities of the Third Age; UTA “-”, seniors not participating in classes at Universities of the Third Age; n, number of participants; *χ*^2^, value of the Chi^2^ statistic; df, degrees of freedom; *p*, level of statistical significance.

Almost 40% (107; 38.63%) of seniors who did not attend classes at the Universities of the Third Age showed a high level of stress, and every fourth (89; 25.14%) Silesian senior taking part in the above-mentioned activity had a low level of stress. The differences observed between the compared groups in the numbers of individual levels of stress intensity turned out to be statistically significant (*p* = 0.04). Therefore, it can be concluded that participation in the discussed activity will contribute to reducing the level of stress felt.

Comparison tests for many independent groups performed using the Kruskall-Wallis ANOVA test did not show statistically significant differences in the number of points obtained by seniors in the PSS-10 questionnaire depending on attendance at Universities of the Third Age, sex, level of education, participants ‘age and place of residence.

**Figure 3:**
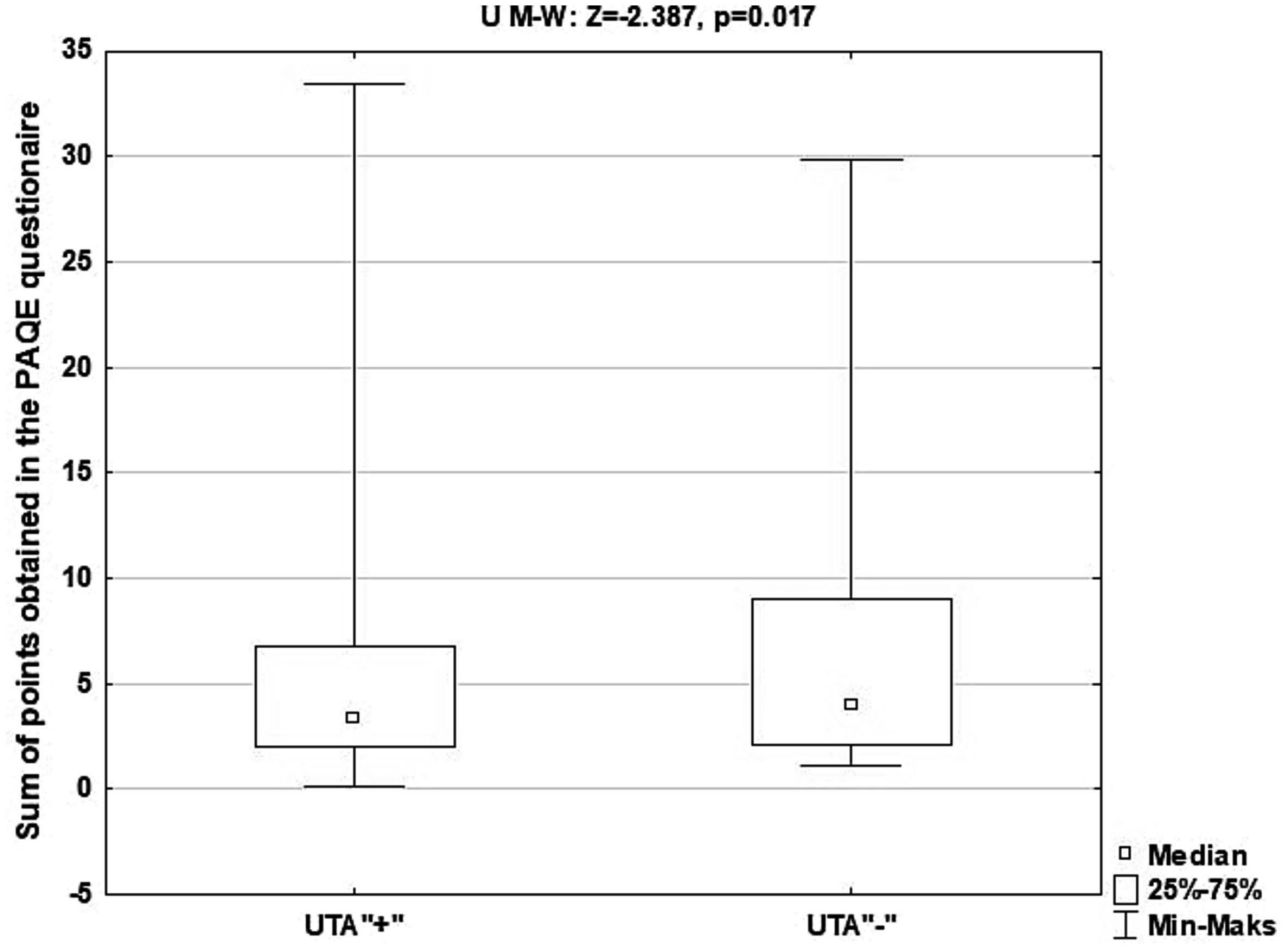
Characteristics of the study group, taking into account the results of the analysis of differences in the total number of points obtained in the PAQE questionnaire by seniors attending classes at the University of the Third Age and denying the above-mentioned activity.

Figure 3 shows the characteristics of the study group, taking into account the results of the analysis of differences in the total number of points obtained in the physical activity assessment questionnaire for seniors (PAQE) by those attending classes at the Universities of the Third Age and those denying described activity, and in Supplementary Table S8 - taking into account attendance or not for classes within Universities of the Third Age, sex, age of participants, level of education and place of residence.

The median of points obtained on the physical activity assessment scale (PAQE) by seniors attending classes at Universities of the Third Age was statistically significantly higher than seniors who denied participation in the mentioned activity (*p* = 0.017). Therefore, it can be concluded that seniors taking part in University of the Third Age activities were in better physical condition than those who did not participate in the above-mentioned activity.

The Kruskal-Wallis test also showed statistically significant (*H* = 11.09; *p* = 0.011) differences in the number of points obtained in the PAQE questionnaire by women participating in classes at Universities of the Third Age, compared to men not participating (*p* = 0.015) in the activity in question. The observed decrease in the median of points obtained could be related to the respondents’ attitude toward engaging in physical activity, depending on attending classes at Universities of the Third Age. The results of the described analysis are presented in Supplementary Figure S4, and the results of multiple comparisons are presented in Supplementary Table S9.

The Kruskal-Wallis test showed statistically significant differences in the number of points obtained in the PAQE physical activity questionnaire (*p* < 0.001). Seniors aged 60 to 70 who attended classes at Universities of the Third Age obtained statistically significantly higher results compared to seniors aged 71 to 80 attending (*p* = 0.013) and not attending (*p* < 0.001) and seniors aged ≥81 years (*p* = 0.001) not attending for classes at Universities of the Third Age. The observed decrease in the median number of points obtained was most likely related to the general deterioration of the health and physical fitness of the surveyed seniors as their age increased. The results of the described analysis are presented in Supplementary Figure S5, and the results of multiple comparisons are presented in Supplementary Table S10.

The analysis also showed a statistically significant (*H* = 38.848; *p* < 0.001) difference between the number of points obtained in the PAQE questionnaire among seniors with secondary education attending (*p* = 0.029) and non-participating (*p* = 0.007) and higher education attending classes at Universities of the Third Age, compared to seniors with primary education who did not participate in the discussed activity. The observed increase in the median number of points along with the increase in the level of education and additional participation in activities at Universities of the Third Age could be related to the frequent provision of information about a proper lifestyle during the activity in question. The results of this analysis are presented in Supplementary Figure S6, and the results of multiple comparisons are presented in Supplementary Table S11.

The Kruskal-Wallis test comparing the number of points obtained in the PAQE physical activity questionnaire between the respondents did not show statistically significant (*H* = 7.720; *p* = 0.05) differences in the number of points obtained by seniors living in cities participating and not participating in the activities of the Universities of the Third Age. The observed lack of a statistically significant difference may be related to the small group of participants, and its enlargement may contribute to obtaining a statistically significant result and further conclusions in the compared groups. The results of the mentioned analysis are presented in Supplementary Figure S7, and the results of multiple comparisons are presented in Supplementary Table S12.

**Figure 4:**
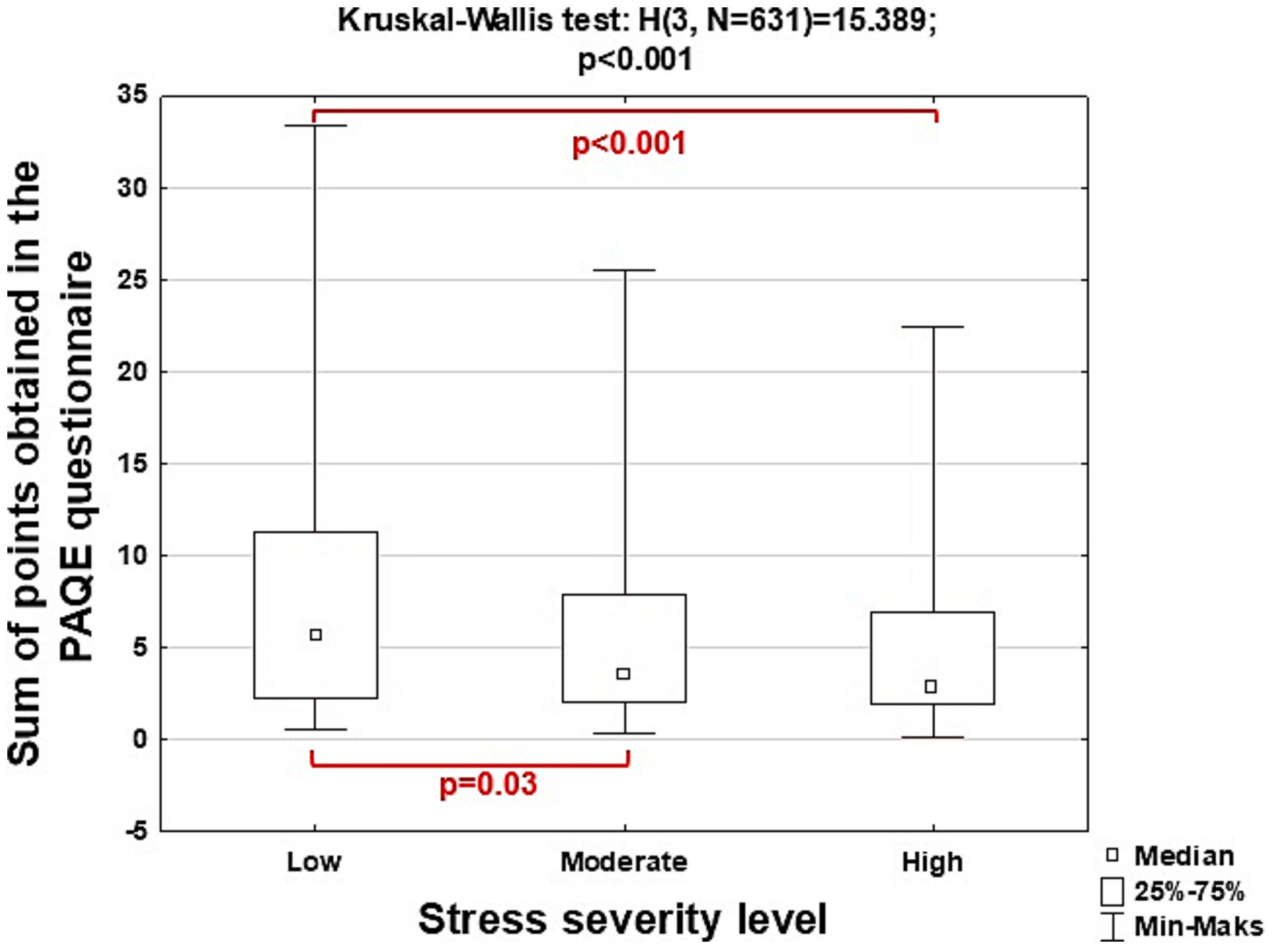
Characteristics of seniors taking into account the results of the analysis of differences in the total number of points obtained in the PAQE questionnaire depending on the level of stress intensity.

Figure 4 shows the characteristics of the study group, including the analysis of differences in the number of points obtained in the PAQE questionnaire and the level of stress in seniors.

The Kruskal-Wallis test did not show statistically significant difference (*H* = 3.945; *p* = 0.14) in the number of points obtained by seniors in the PAQE physical activity questionnaire depending on the severity of depression measured using the Geriatric Depression Rating Scale questionnaire. Moreover, the mentioned test showed statistically significant differences between the intensity of stress examined using the PSS-10 questionnaire and the points obtained by the respondents when assessing physical activity with the PAQE questionnaire (*H* = 15.389; *p* = <0.001). Post-hoc analysis showed statistically significant differences in the number of points obtained by seniors with low versus moderate (*p* = 0.03) and low versus high (*p* < 0.001) levels of stress intensity. The observed differences can be explained by the fact that the increased physical activity of the surveyed seniors reduced stress.

## Discussion

In our study, almost 40% (141; 39.83%) of seniors participating in activities at Universities of the Third Age did not show signs of depression, while severe depression was observed in 11 (3.97%) seniors who denied participating in the mentioned activity, and the mentioned differences were statistically significant (*p* = 0.004). Older adult depression is a popular and difficult to diagnose condition. It is often confused with dementia (14). However, research shows that seniors cared for by the environment around them are less likely to experience symptoms of depression than those left to themselves (15). Attending classes at Universities of the Third Age allows seniors to develop social contacts with peers and awakens their sense of “being needed,” which has a positive impact on their well-being and quality of life (8). This was also confirmed by our own research, where a statistically significantly higher score on the Geriatric Depression Rating Scale according to Yesavage, compared to respondents confirming their participation in the above-mentioned activity (*p* = 0.002), which proves that participation in University of the Third Age activities had a positive impact on well-being and reduced the risk of developing depression by maintaining social contacts. In their research, I. Wróblewska and J. Błaszczyk showed that the most common motive for taking up activities at Universities of the Third Age by seniors was the desire to expand their knowledge and maintain intellectual fitness (93; 81.58%), and for more than half (63; 55 0.26%) – maintaining bonds with people from the same age group (16).

In her work on the contemporary needs of Universities of the Third Age, M. Sulik quoted a student of the University of Third Age in Katowice who stated that “*loneliness is a paralyzing factor and it is highly advisable to seek new acquaintances or contacts with nice people with similar interests. Such opportunities were offered by the UTA, and belonging to this group of students triggered new interests in me, stimulated my activity, and allowed me to discover interesting places in the immediate vicinity and throughout our country*” (17). Based on observations in our study, it can also be concluded that participation in classes at Universities of the Third Age had a positive impact on reducing the level of depression, especially among “younger” seniors (over 60 years of age), educated and those living in cities where access to the offer of classes conducted by Universities of the Third Age is much larger (18).

Our study involving a group of Silesian seniors attending classes at the University of the Third Age showed a statistically significantly lower score on the PSS-10 stress scale compared to the results of the surveyed seniors who denied participation in the mentioned activity (*p* = 0.04). Almost 40% (107; 38.63%) of seniors who did not attend classes at Universities of the Third Age showed a high level of stress, while every fourth (89; 25.14%) senior who took part in the above-mentioned activity declared a low level of stress - the mentioned differences were also statistically significant (*p* = 0.04). Stress is one of the main factors determining overall life satisfaction. J. Czapiński confirmed this in his research, in which he showed that the occurrence of stressful situations negatively affects human mental health (19). Pawlikowska-Łagód K. et al. showed that the general satisfaction of seniors with life deteriorates as the level of stress increases (20). Stress also influences the development of depression (14). Many universities also organize classes on techniques for dealing with stressful situations, and research shows that seniors attending classes at Universities of the Third Age are better able to cope with stress (21).

According to the study “*Gender differences in mental disorders and suicide in Europe*” conducted in 2014 in cooperation with European universities, women were more exposed to stress and coped with it worse than men. According to the cited studies, this is a direct result of more responsibilities imposed on women in connection with professional work and running the house - as if they worked two jobs, thus providing themselves with a large dose of stress (22, 23). Our study involved retired seniors, so no statistically significant differences were found in the number points obtained by seniors in the PSS-10 questionnaire depending on gender, but also level of education, age and place of residence. There are reports in the literature that living in the countryside is associated with a low level of stress (24, 25), and working in positions requiring higher education - with a high level of stress (26, 27). However, the mentioned studies were conducted in groups of people of working age, not among seniors.

B. Lejzerowicz-Zajązkowska and P. Hajduk conducted an analysis of the aging society, in which they showed that physical activity had a significant impact on increasing independence and self-reliance, and thus improved the quality of life of older people (28). In our study, the median of points obtained on the physical activity assessment scale (PAQE) by the surveyed seniors participating in the activities of the Universities of the Third Age was statistically significantly higher than the respondents who denied participation in the discussed activity (*p* = 0.017), meaning that the seniors participating in the activities were more physically active, than the rest. Universities of the Third Age organize many activities related to physical activity for seniors contributing to improvement of quality of their health and life (8).

Seniors attending the above-mentioned classes are also better educated about not only the positive impact of physical activity on the body, but also the principles of leading a healthy lifestyle than seniors who do not participate in them (8). Our study also confirmed a statistically significant (*H* = 38.848; *p* < 0.001) differences between the number of points obtained in the PAQE questionnaire among seniors and their level of education. The observed increase in the median number of points along with the increase in the level of education and participation in activities at Universities of the Third Age could be related to the frequent provision of information about a proper lifestyle during the activity in question.

Our study also compared the number of points obtained in the PAQE physical activity questionnaire and place of residence showed no statistically significant differences in the number of points obtained by seniors living in cities participating and not participating in the activities of the Universities of the Third Age. This showed that seniors living in cities and taking part in UTA activities are not more physically active than those who do not take part in the above-mentioned activity.

Our study showed that women attending University of the Third Age classes were significantly more likely to engage in physical activity compared to men who did not participate in the above-mentioned classes. K. Witkowski et al. examined the physical activity of women over 55 years of age, and their research showed that 111 (93%) of the respondents systematically practiced some form of physical activity, and the most popular of them were walking (38; 31%) and Nordic walking (31; 26%) (29). Women also seem to be more motivated to participate in the activities of Universities of the Third Age, as evidenced by statistics: according to the report of the Department of Social Research and Living Conditions of the Statistical Office in Gdańsk (Poland), women constituted as much as 86.00% of all students of Universities of the Third Age (30). Therefore, they are more likely to take part in activating activities and translate positive habits related to regular physical activity into everyday life.

A statistically significant reduction was observed in the number of seniors aged ≥81 years (H = 35.93; *p* < 0.001) engaging in physical activity and not attending University of the Third Age classes compared to “younger” seniors participating in the above-mentioned activity (*p* = 0.001). According to the previously cited report of the Department of Social Research and Living Conditions of the Statistical Office in Gdańsk, the largest age group, comprising 59.7% of all UTA students, were people aged 60 to 70, and people over 76 years of age only 11% (30). The reduction in the number of “older” participants in classes offered by Universities of the Third Age, as well as the reduction in their level of physical activity, was most likely related to the general deterioration of the health and physical fitness of the surveyed seniors with age.

In their research on the importance of physical activity in the prevention of depressive disorders, T. Saran et al. came to the conclusion that in the absence of health contraindications, regular physical exercise can be a form of prevention of depressive disorders, as well as a technique complementing pharmacological treatment and psychotherapy of depressive disorders (31). J. Tackas also mentioned the important role of physical activity in the prevention and treatment of depressive disorders (32). K. Melnik et al. confirmed that physical activity may affect mental health, and potentially also the risk of developing various mental disorders, and may even serve as a method of treating them (33).

Statistically significant differences were found between the intensity of stress examined using the PSS-10 questionnaire and the points obtained by seniors in the assessment of physical activity using the PAQE questionnaire (*H* = 15.389; *p* < 0.001). There were also statistically significant differences in the number of points obtained by respondents with low versus moderate (*p* = 0.03) and low versus high (*p* < 0.001) levels of stress intensity, which could be explained by the fact that the increased physical activity of the surveyed seniors resulted in a reduction of stress. Many researchers have previously obtained similar results showing the beneficial effect of physical activity on reducing stress levels. Examples include the research of K. Zając et al., in which a significant reduction in the level of perceived stress was noted as a result of regular participation in organized group general gymnastics classes in a group of women over 60 years of age (34), or M. Lipko-Kowalska, who, researching physical activity of women of mature age, in her work she presented, among others, the most common reasons for undertaking the above-mentioned activity by the respondents, and these were: the desire to maintain health, not only physical, but also mental (35). Studies conducted in other groups of respondents also confirmed the discussed results (36–38). According to many reports already cited in the initial report, physical activity allows, among other things, to reduce stress and contributes to improved well-being (4–7). At this stage, the limitations of the study include a relatively small group of seniors participating in University of the Third Age activities. Moreover, there were definitely more women, seniors aged 61–70, as well as seniors living in urban areas, where there is a greater opportunity to choose UTA classes, however the last factor may have a misleading impact on seniors’ attendance at UTA classes. Additionally, all surveyed seniors lived in the same area of the Silesian agglomeration. Nevertheless, this study showed the relationship between leading a healthy lifestyle, as well as the reduction or complete absence of symptoms of depression and stress in the group of seniors with their participation in the discussed activities of the Universities of the Third Age, and also gave an insight into the possibility of preventing the mentioned symptoms thanks to the integration of older people and satisfying their social and intellectual needs. To conclude all obtained and discussed result it can be stated that better well-being and stress reduction of the surveyed seniors, as well as their greater physical activity, were associated with participation in activities at the Universities of the Third Age. Our study proves that activities undertaken by seniors have a real impact on their life and wellbeing. Our study also proves that it is important to promote and start actions leading to seniors’ better inclusion to the society life especially those influencing their health-promoting habits and helping to maintain their focus and memory functions. This study is a prove for municipal authorities that implementation of discussed actions can slow down the health deterioration and help to preserve good aging of different regions inhabitants. Moreover, it could have positive impact not only for seniors but also people meeting with them who can take from their knowledge and experience. Future research should concentrate on reasons why many seniors do not attend activities in their leisure time - especially on accessibility of various activities and financial reasons, which in the future will play crucial role in the aging societies.

## Data availability statement

The original contributions presented in the study are included in the article/Supplementary material, further inquiries can be directed to the corresponding author.

## Ethics statement

The research began after obtaining the consent of the Bioethics Committee of the Medical University of Silesia in Katowice - resolution no. PCN/0022/KB1/36/21. The studies were conducted in accordance with the local legislation and institutional requirements. The participants provided their written informed consent to participate in this study.

## Author contributions

JD: Conceptualization, Investigation, Methodology, Project administration, Software, Supervision, Validation, Visualization, Writing – original draft, Writing – review & editing. MS: Data curation, Formal analysis, Methodology, Resources, Validation, Writing – original draft, Investigation, Writing – review & editing. EŁ: Data curation, Methodology, Writing – original draft. OS: Formal analysis, Resources, Writing – original draft, Validation, Writing – review & editing.
