# Supplementary material for: Selected elements of the lifestyle of Silesian seniors, taking into account their participation in the activities of the Third Age Universities
Source: Front Public Health. 2024 Mar 4;12:1375238. doi: 10.3389/fpubh.2024.1375238 (PMC10946672; doi:10.3389/fpubh.2024.1375238)
Supplement: Supplementary file 1 [file Data_Sheet_1.docx]

**Supplementary material for article:**

**Selected elements of the lifestyle of Silesian seniors, taking into account their participation in the activities of the Third Age Universities**

**Tab. ST1.** Characteristics of the studied group of seniors, including descriptive statistics of the scores obtained by respondents in the Yesavage Geriatric Depression Assessment Questionnaire

| **Seniors’ studied group (n=631; 100%)** | | |
| --- | --- | --- |
| **Variables** | **UTA „+”** | **UTA „-”** |
| **Mean value** | 6.19 | 6.68 |
| **Standard deviation** | 1.64 | 1.79 |
| **Median** | 6 | 6 |
| **Min.** | 2 | 2 |
| **Max.** | 11 | 13 |
| **Lover quartile** | 5 | 5 |
| **Upper quartile** | 7 | 8 |

**Explanation of abbreviations:** UTA "+" - seniors participating in Third Age Universities activities, UTA "-" - seniors refusing to attend classes at Universities of the Third Age, Min. - minimum value, Max. – maximum value

**Tab.** **ST2.** Characteristics of the studied group of seniors, including descriptive statistics regarding the points obtained in the Yesavages Geriatric Depression Rating Scale questionnaire and attending classes at Universities of the Third Age, gender, age, education and place of residence

| **Seniors’ studied group (n=631; 100%)** | | | | | | | | | | | | |
| --- | --- | --- | --- | --- | --- | --- | --- | --- | --- | --- | --- | --- |
| **Variables** | | | **Size of a given group (n; %)** | | **Descriptive statistics of the sum of points obtained in the Yesavage’s Geriatric Depression Rating Scale questionnaire** | | | | | | | |
|  |  |  | **n** | **%** | $\bar{\boldsymbol{x}}$ | **SD** | **Median** | **Min.** | **Max.** | **Q1** | | **Q3** |
| **Sex** | **Women** | UTA”+” | 303 | 48.02 | 6.23 | 1.67 | 6.00 | 2.00 | 11.00 | 5.00 | 7.00 | |
|  |  | UTA”-” | 172 | 27.26 | 6.67 | 1.84 | 6.00 | 2.00 | 13.00 | 5.00 | 8.00 | |
|  | **Women** | UTA”+” | 51 | 8.08 | 5.96 | 1.43 | 5.00 | 3.00 | 9.00 | 5.00 | 7.00 | |
|  |  | UTA”-” | 105 | 16.64 | 6.69 | 1.70 | 6.00 | 4.00 | 12.00 | 5.00 | 8.00 | |
| **Age**  **[years]** | **60-70** | UTW”+” | 216 | 34,23 | 6,17 | 1,61 | 6,00 | 2,00 | 11,00 | 5,00 | 7,00 | |
|  | **60-70** | UTA”-” | 157 | 24,88 | 6,36 | 1,68 | 6,00 | 4,000 | 13,00 | 5,00 | 7,00 | |
|  | **71-80** | UTA”+” | 116 | 18,38 | 6,31 | 1,75 | 6,00 | 3,00 | 11,00 | 5,00 | 7,00 | |
|  | **71-80** | UTA”-” | 104 | 16,48 | 7,10 | 1,87 | 7,00 | 2,000 | 11,00 | 6,00 | 8,00 | |
|  | **≥81** | UTA”+” | 22 | 3,49 | 5,77 | 1,34 | 6,00 | 3,00 | 8,00 | 5,00 | 7,00 | |
|  | **≥81** | UTA”-” | 16 | 2,54 | 7,06 | 1,692 | 7,00 | 5,00 | 12,00 | 6,00 | 7,50 | |
| **Level of education** | **Primary** | UTA”+” | 0 | 0.00 | 0 | 0 | 0 | 0 | 0 | 0 | 0 | |
|  |  | UTA”-” | 37 | 5.86 | 7.22 | 1.67 | 7.00 | 5.00 | 11.00 | 6.00 | 8.00 | |
|  | **Vocational** | UTA”+” | 27 | 4.28 | 5.89 | 1.19 | 6.00 | 3.00 | 8.00 | 5.00 | 6.00 | |
|  |  | UTA”-” | 93 | 14.74 | 6.89 | 1.82 | 6.00 | 4.00 | 11.00 | 6.00 | 8.00 | |
|  | **Secondary** | UTA”+” | 195 | 30.90 | 22.88 | 6.72 | 22.00 | 10.30 | 52.35 | 18.47 | 26.87 | |
|  |  | UTA”-” | 97 | 15.37 | 6.49 | 1.78 | 6.00 | 2.00 | 12.00 | 5.00 | 7.00 | |
|  | **Higher** | UTA”+” | 132 | 20.92 | 6.5 | 1.48 | 6.00 | 3.00 | 10.00 | 5.00 | 7.00 | |
|  |  | UTA”-” | 50 | 7.92 | 6.22 | 1.71 | 6.00 | 4.00 | 13.00 | 5.00 | 7.00 | |
| **Place of residence** | **City** | UTA”+” | 338 | 53.57 | 6.19 | 1.63 | 6.00 | 2.00 | 11.00 | 5.00 | | 7.00 |
|  |  | UTA”-” | 98 | 15.53 | 6.76 | 1.96 | 6.00 | 4.00 | 12.00 | 5.00 | | 8.00 |
|  | **Countryside** | UTA”+” | 16 | 2.54 | 6.25 | 1.98 | 6.00 | 3.00 | 11.00 | 5.00 | | 7.00 |
|  |  | UTA”-” | 179 | 28.37 | 6.63 | 1.69 | 6.00 | 2.00 | 13.00 | 5.00 | | 8.00 |

**Explanation of abbreviations:** n – size of a given group, $\bar{x}$ – mean value, SD – standard deviation, Min. – minimum value, Max. – maximum value, Q1 – lower quartile, Q3 – upper quartile

**Explanation of abbreviations:** A - seniors aged 61 to 70 attending classes at Universities of the Third Age, B - seniors aged 61 to 70 not attending classes at Universities of the Third Age, C - seniors aged 71 to 80 attending classes at Universities of the Third Age, D - seniors aged 71 to 80 not attending classes at Universities of the Third Age, E - seniors aged ≥81 years old attending classes at Universities of the Third Age, F - seniors aged ≥81 years not attending classes at Universities of the Third Age, H - value of the Kruskall-Wallis ANOVA test, p - level of statistical significance

**Fig. SF1.** Characteristics of the study group, including the number of points obtained in the Yesavage’s Geriatric Depression Rating Scale questionnaire, attending classes at Universities of the Third Age and the age of the surveyed seniors

**Tab. ST3.** The value of statistical significance for multiple comparisons of the sum of points obtained in the Yeavage’s Geriatric Depression Rating Scale questionnaire according to Yesavage, taking into account the age of the surveyed seniors and participation in activities of Universities of the Third Age

| **p-value for multiple comparisons Kruskal-Wallis test: H(7, N= 631)=25,815 p<0,001** | | | | | | |
| --- | --- | --- | --- | --- | --- | --- |
| **The sum of points obtained in the Yesavage’s Geriatric Gepression Rating Scale questionnaire** | **A** | **B** | **C** | **D** | **E** | **F** |
| **A** | - | NS | NS | **<0,01** | NS | NS |
| **B** | NS | - | NS | **0,008** | NS | NS |
| **C** | NS | NS | - | **0,020** | NS | NS |
| **D** | **<0,01** | **0,008** | **0,020** | - | NS | NS |
| **E** | NS | NS | NS | NS | - | NS |
| **F** | NS | NS | NS | NS | NS | - |

**Explanation of abbreviations:** A - seniors aged 60 to 70 years attending classes at Universities of the Third Age, B - seniors aged 60 to 70 years not attending classes at Universities of the Third Age, C - seniors aged 71 to 80 years who attend classes at Universities of the Third Age, D - seniors aged 71 to 80 years who do not attend classes at Universities of the Third Age, E - seniors aged ≥81 years attending classes at Universities of the Third Age, F - seniors aged ≥81 years not attending classes at Universities of the Third Age, NS - no statistical significance, H - Kruskall-Wallis ANOVA test value, p - level of statistical significance. Red font in the supplementary tables indicates statistical significance.

**Explanation of abbreviations:** P-UTA”-” – seniors with primary education not attending classes at Universities of the Third Age, Z- UTA”+” – seniors with vocational education attending classes at Universities of the Third Age, Z- UTA”-” – seniors with education professional who do not attend classes at Universities of the Third Age, S- UTA "+" - seniors with secondary education attending classes at Universities of the Third Age, S- UTA "-" - seniors with secondary education not attending classes at Universities of the Third Age, W- UTA "+" – seniors with higher education attending classes at Universities of the Third Age, W- UTA”-” – seniors with higher education not attending classes at Universities of the Third Age, H – value of the Kruskall-Wallis ANOVA test, p – level of statistical significance

**Fig. SF2.** Characteristics of the study group, including the number of points obtained in the Yesavage’s Geriatric Depression Rating Scale questionnaire and attending classes at Universities of the Third Age and level of education

**Tab. ST4.** The value of statistical significance for multiple comparisons of the sum of points obtained in the Yesavage’s Geriatric Depression Rating Scale questionnaire, taking into account level of education and participation in classes at Universities of the Third Age

| **p-value for multiple comparisons**  **Kruskal-Wallis test: H(7,N= 631)=23.655, p=0.0006** | | | | | | | |
| --- | --- | --- | --- | --- | --- | --- | --- |
| **The sum of points obtained in the Yesavage’s Geriatric Gepression Rating Scale questionnaire** | **P- UTA”-”** | **Z- UTA”+”** | **Z- UTA”-”** | **S- UTA”+”** | **S- UTA”-”** | **W- UTA”+”** | **W- UTA”-”** |
| **P- UTA”-”** | - | NS | NS | NS | NS | **0.01** | NS |
| **Z- UTA”+”** | NS | - | NS | NS | NS | NS | NS |
| **Z- UTA”-”** | NS | NS | - | NS | NS | **0.02** | NS |
| **S- UTA”+”** | NS | NS | NS | - | NS | NS | NS |
| **S- UTA”-”** | NS | NS | NS | NS | - | NS | NS |
| **W- UTA”+”** | **0.01** | NS | **0.02** | NS | NS | - | NS |
| **W- UTA”-”** | NS | NS | NS | NS | NS | NS | - |

**Wyjaśnienie skrótów:** P-UTA”-” – seniors with primary education not attending classes at Universities of the Third Age, Z- UTA”+” – seniors with vocational education attending classes at Universities of the Third Age, Z- UTA”-” – seniors with education professional who do not attend classes at Universities of the Third Age, S- UTA "+" - seniors with secondary education attending classes at Universities of the Third Age, S- UTA "-" - seniors with secondary education not attending classes at Universities of the Third Age, W- UTA "+" – seniors with higher education attending classes at Universities of the Third Age, W- UTA”-” – seniors with higher education not attending classes at Universities of the Third Age, H – value of the Kruskall-Wallis ANOVA test, p – level of statistical significance, NS – non significant. Red font in the supplementary tables indicates statistical significance.

**Explanation of abbreviations:** H - value of the Kruskall-Wallis ANOVA test, p - level of statistical significance, 1 - seniors living in the city, attending classes at Universities of the Third Age, 2 - seniors living in the city, not attending classes at Universities of the Third Age, 3 - seniors living in the countryside, attending classes at Universities of the Third Age, 4 - seniors living in the countryside, not attending classes at Universities of the Third Age

**Fig. SF3.** Characteristics of the study group, including the number of points obtained in the Yesavage’s Geriatric Depression Rating Scale questionnaire and attending classes at Universities of the Third Age and place of residence

**Tab. ST5.** The value of statistical significance for multiple comparisons of the sum of points obtained in the Yeavage’s Geriatric Depression Rating Scale questionnaire according to Yesavage, taking into account the place of residence of the surveyed seniors and participation in activities of Universities of the Third Age

| **p-value for multiple comparisons**  **Kruskal-Wallis test: H(3,N= 631)=10.529 p=0.015** | | | | |
| --- | --- | --- | --- | --- |
| **The sum of points obtained in the Yesavage’s Geriatric Gepression Rating Scale questionnaire** | **1** | **2** | **3** | **4** |
| **1** | - | NS | NS | **0.03** |
| **2** | NS | - | NS | NS |
| **3** | NS | NS | - | NS |
| **4** | **0.03** | NS | NS | - |

**Explanation of abbreviations:** H - value of the Kruskall-Wallis ANOVA test, p - level of statistical significance, 1 - seniors living in the city, attending classes at Universities of the Third Age, 2 - seniors living in the city, not attending classes at Universities of the Third Age, 3 - seniors living in the countryside, attending classes at Universities of the Third Age, 4 - seniors living in the countryside, not attending classes at Universities of the Third Age, NS - no statistical significance. Red font in the supplementary tables indicates statistical significance.

**Tab. ST6.** Characteristics of the study group, including descriptive statistics of the scores obtained by respondents in the PSS-10 stress assessment questionnaire

| **Variables** | **Data** | |
| --- | --- | --- |
|  | **UTA „+”** | **UTA „-”** |
| **Mean value** | 16.9 | 17.94 |
| **Standard deviation** | 5.16 | 5.14 |
| **Median** | 17 | 18 |
| **Min.** | 4 | 4 |
| **Max.** | 31 | 32 |
| **Lover quartile** | 13 | 14 |
| **Upper quartile** | 20 | 21 |

**Explanation of abbreviations:** UTA "+" - seniors participating in Third Age Universities activities, UTA "-" - seniors refusing to attend classes at Universities of the Third Age, Min. - minimum value, Max. – maximum value

**Tab. ST7.** Characteristics of the surveyed group of seniors, including descriptive statistics regarding the points obtained in the stress assessment questionnaire - PSS-10, and attendance at classes at Universities of the Third Age, gender, age, education and place of residence

| **Seniors’ studied group (n=631; 100%)** | | | | | | | | | | | |
| --- | --- | --- | --- | --- | --- | --- | --- | --- | --- | --- | --- |
| **Zmienne** | | | **Size of a given group (n; %)** | | **Descriptive statistics of the sum of points obtained in the stress assessment questionnaire – PSS-10** | | | | | | |
|  |  |  | **n** | **%** | $\bar{\boldsymbol{x}}$ | **SD** | **Mediana** | **Min.** | **Max.** | **Q1** | **Q3** |
| **Sex** | **Women** | UTA”+” | 303 | 48.02 | 17.04 | 5.14 | 17.00 | 4.00 | 31.00 | 13.00 | 20.00 |
|  |  | UTA”-” | 172 | 27.26 | 17.87 | 5.48 | 18.00 | 4.00 | 32.00 | 14.00 | 21.00 |
|  | **Women** | UTA”+” | 51 | 8.08 | 16.08 | 5.21 | 17.00 | 4.00 | 29.00 | 13.00 | 19.00 |
|  |  | UTA”-” | 105 | 16.64 | 18.04 | 18.00 | 6.00 | 29.00 | 15.00 | 21.00 | 4.55 |
| **Age**  **[years]** | **60-70** | UTA”+” | 216 | 34,23 | 6,18 | 1,61 | 6,00 | 2,00 | 11,00 | 5,00 | 7,00 |
|  | **60-70** | UTA”-” | 157 | 24,88 | 17,38 | 5,24 | 17,00 | 4,00 | 32,00 | 14,00 | 20,00 |
|  | **71-80** | UTA”+” | 116 | 18,38 | 17,18 | 4,823 | 17,50 | 4,00 | 27,00 | 14,00 | 20,00 |
|  | **71-80** | UTA”-” | 104 | 16,48 | 18,84 | 5,034 | 19,00 | 6,000 | 32,00 | 15,00 | 22,00 |
|  | **≥81** | UTA”+” | 22 | 3,49 | 16,32 | 3,884 | 16,00 | 10,00 | 23,00 | 14,00 | 19,00 |
|  | **≥81** | UTA”-” | 16 | 2,54 | 17,56 | 4,163 | 17,50 | 12,00 | 28,00 | 14,50 | 19,50 |
| **Level of education** | **Primary** | UTA”+” | 0 | 0.00 | 0 | 0 | 0 | 0 | 0 | 0 | 0 |
|  |  | UTA”-” | 37 | 5.86 | 18.81 | 5.43 | 19.00 | 4.00 | 29.00 | 15.00 | 22.00 |
|  | **Vocational** | UTA”+” | 27 | 4.28 | 17.07 | 4.39 | 17.00 | 11.00 | 26.00 | 14.00 | 20.00 |
|  |  | UTA”-” | 93 | 14.74 | 17.56 | 5.08 | 18.00 | 6.00 | 31.00 | 14.00 | 20.00 |
|  | **Secondary** | UTA”+” | 195 | 30.90 | 6.33 | 1.79 | 6.00 | 2.00 | 11.00 | 5.00 | 7.00 |
|  |  | UTA”-” | 97 | 15.37 | 18.28 | 5.28 | 18.00 | 6.00 | 32.00 | 15.00 | 21.00 |
|  | **Higher** | UTA”+” | 132 | 20.92 | 16.27 | 5.21 | 16.00 | 4.00 | 29.00 | 12.50 | 20.00 |
|  |  | UTA”-” | 50 | 7.92 | 17.32 | 4.76 | 17.00 | 5.00 | 28.00 | 15.00 | 20.00 |
| **Place of residence** | **City** | UTA”+” | 338 | 53.57 | 16.87 | 5.04 | 17.00 | 4.00 | 29.00 | 14.00 | 20.00 |
|  |  | UTA”-” | 98 | 15.53 | 18.49 | 5.91 | 18.00 | 5.00 | 32.00 | 15.00 | 22.00 |
|  | **Countryside** | UTA”+” | 16 | 2.54 | 17.56 | 7.42 | 18.50 | 6.00 | 31.00 | 11.50 | 23.50 |
|  |  | UTA”-” | 179 | 28.37 | 17.63 | 4.66 | 18.00 | 4.00 | 30.00 | 14.00 | 2100 |

**Explanation of abbreviations:** n – size of a given group, $\bar{x}$ – mean value, SD – standard deviation, Min. – minimum value, Max. – maximum value, Q1 – lower quartile, Q3 – upper quartile.

**Tab. ST8.** Characteristics of the surveyed group of seniors, including descriptive statistics regarding the points obtained in the PAQE physical activity assessment questionnaire and attending classes at Universities of the Third Age, gender, age, education and place of residence

| **Seniors’ studied group (n=631; 100%)** | | | | | | | | | | | |
| --- | --- | --- | --- | --- | --- | --- | --- | --- | --- | --- | --- |
| **Zmienne** | | | **Size of a given group (n; %)** | | **Descriptive statistics of the sum of points obtained in the PAQE physical activity assessment questionnaire** | | | | | | |
|  |  |  | **n** | **%** | $\bar{\boldsymbol{x}}$ | **SD** | **Mediana** | **Min.** | **Max.** | **Q1** | **Q3** |
| **Sex** | **Women** | UTA”+” | 303 | 48.02 | 6.16 | 6.16 | 4.02 | 1.10 | 29.8 | 2.20 | 9.27 |
|  |  | UTA”-” | 172 | 2726 | 5.41 | 5.19 | 3.39 | 0.10 | 31.1 | 2.06 | 7.27 |
|  | **Women** | UTA”+” | 51 | 8.08 | 5.04 | 4.27 | 3.14 | 1.20 | 18.0 | 1.70 | 7.27 |
|  |  | UTA”-” | 105 | 16.64 | 4.82 | 4.92 | 3.30 | 0.30 | 33.4 | 1.60 | 6.43 |
| **Age**  **[years]** | **60-70** | UTA”+” | 216 | 34,23 | 6.91 | 5.64 | 4.73 | 1.10 | 29.8 | 2.24 | 10.5 |
|  | **60-70** | UTA”-” | 157 | 24,88 | 5.96 | 5.48 | 4.08 | 0.30 | 31.1 | 2.26 | 7.99 |
|  | **71-80** | UT”+” | 116 | 18,38 | 4.66 | 3.59 | 3.10 | 1.10 | 16.2 | 1.85 | 6.50 |
|  | **71-80** | UTA”-” | 104 | 16,48 | 4.37 | 4.48 | 2.69 | 0.10 | 33.4 | 1.75 | 5.33 |
|  | **≥81** | UTA”+” | 22 | 3,49 | 4.09 | 3.27 | 2.43 | 1.10 | 13.1 | 2.10 | 6.40 |
|  | **≥81** | UTA”-” | 16 | 2,54 | 2.89 | 3.19 | 1.74 | 0.40 | 12.7 | 1.10 | 3.01 |
| **Level of education** | **Primary** | UTA”+” | 0 | 0.00 | 0.00 | 0.00 | 0.00 | 0.00 | 0.00 | 0.00 | 0.00 |
|  |  | UTA”-” | 37 | 5.86 | 3.21 | 2.87 | 2.02 | 0.80 | 12.7 | 1.60 | 3.31 |
|  | **Vocational** | UTA”+” | 27 | 4.28 | 4.16 | 4.47 | 2.30 | 1.10 | 20.2 | 1.50 | 5.12 |
|  |  | UTA”-” | 93 | 14.74 | 4.67 | 4.96 | 2.68 | 0.30 | 31.1 | 1.80 | 5.58 |
|  | **Secondary** | UTA”+” | 195 | 30.90 | 5.46 | 4.67 | 3.33 | 1.10 | 29.8 | 2.10 | 8.56 |
|  |  | UTA”-” | 97 | 15.37 | 6.34 | 6.09 | 4.38 | 0.10 | 33.4 | 2.10 | 8.72 |
|  | **Higher** | UTA”+” | 132 | 20.92 | 7.17 | 5.49 | 6.27 | 1.10 | 25.9 | 2.41 | 10.2 |
|  |  | UTA”-” | 50 | 7.92 | 5.36 | 3.86 | 4.50 | 0.60 | 21.5 | 2.50 | 7.92 |
| **Place of residence** | **City** | UTA”+” | 338 | 53.57 | 5.93 | 4.95 | 3.99 | 1.10 | 29.8 | 2.10 | 8.88 |
|  |  | UTA”-” | 98 | 15.53 | 4.53 | 3.97 | 2.83 | 0.80 | 21.5 | 1.90 | 5.98 |
|  | **Countryside** | UTA”+” | 16 | 2.54 | 7.36 | 697 | 3.96 | 1.10 | 22.6 | 2.49 | 10.7 |
|  |  | UTA”-” | 179 | 28.37 | 5.54 | 5.59 | 3.56 | 0.10 | 33.4 | 2.00 | 8.04 |

**Explanation of abbreviations:** n – size of a given group, $\bar{x}$ – mean value, SD – standard deviation, Min. – minimum value, Max. – maxium value, Q1 – lower quartile, Q3 – upper quartile

**Explanation of abbreviations:** H - value of the Kruskall-Wallis ANOVA test, p - level of statistical significance, W-UTA"+" - women attending classes at Universities of the Third Age, W-UTA "-" - women not attending classes at Universities of the Third Age, M- UTA”+” – men attending classes at Universities of the Third Age, M- UTA”-” – men not attending classes at Universities of the Third Age.

**Fig. SF4.** Characteristics of the study group, including the number of points obtained in the PAQE questionnaire, attendance at Universities of the Third Age and sex

**Tab. ST9.** The value of statistical significance for multiple comparisons of the sum of points obtained in the PAQE physical activity assessment questionnaire taking into account sex oh the surveyed seniors and participation in activities of Universities of the Third Age

| **p-value for multiple comparisons**  **Kruskal-Wallis test: H(3, N= 631)=11.09; p=0.011** | | | | |
| --- | --- | --- | --- | --- |
| **Sum of points obtained in the PAQE physical activity assessment questionnaire** | **W-UTA”+”** | **W-UTA”-”** | **M-UTA”+”** | **M-UTA”-„** |
| **W-UTA”+”** | - | NS | NS | **0.015** |
| **W-UTA”-”** | NS | - | NS | NS |
| **M-UTA”+”** | NS | NS | - | NS |
| **M-UTA”-„** | **0.015** | NS | NS | - |

**Explanation of abbreviations:** H – value of the Kruskall-Wallis ANOVA test, p – level of statistical significance, W-UTA”+” – women attending classes at Universities of the Third Age, W-UTA”-” – women not attending classes at Universities of the Third Age, M-UTA”+ ” – men attending classes at Universities of the Third Age, M-UTA”-” – men not attending classes at Universities of the Third Age, NS – no statistical significance. Red font in the supplementary tables indicates statistical significance.

.

**Explanation of abbreviations:** A - seniors aged 60 to 70 attending classes Universities of the Third Age, B - seniors aged 60 to 70 not attending classes at Universities of the Third Age, C - seniors aged 71 to 80 attending classes at Universities of the Third Age, D - seniors aged 71 to 80 not attending classes at Universities of the Third Age, E - seniors aged ≥81 years attending Universities of the Third Age classes, F - seniors aged ≥81 years not attending classes at Universities of the Third Age, H - value of the Kruskall-Wallis ANOVA test, p - level of statistical significance.

**Fig. SF5.** Characteristics of the study group, including the number of points obtained in the PAQE questionnaire, attendance at Universities of the Third Age and the age of the surveyed seniors

**Tab. ST10.** The value of statistical significance for multiple comparisons of the sum of points obtained in the PAQE questionnaire, taking into account the age of the surveyed seniors and participation in classes at Universities of the Third Age

| **p-value for multiple comparisons Kruskal-Wallis test: H(3, N= 631)=35.93, p<0.001** | | | | | | |
| --- | --- | --- | --- | --- | --- | --- |
| **Sum of points obtained in the PAQE physical activity assessment questionnaire** | **A** | **B** | **C** | **D** | **E** | **F** |
| **A** | - | NS | **0.013** | **<0.001** | NS | **0.001** |
| **B** | NS | - | NS | NS | NS | **0.013** |
| **C** | **0.013** | NS | - | NS | NS | NS |
| **D** | **<0.001** | NS | NS | - | NS | NS |
| **E** | NS | NS | NS | NS | - | NS |
| **F** | **0.001** | **0.013** | NS | NS | NS | - |

**Explanation of abbreviations:** A - seniors aged 60 to 70 attending classes Universities of the Third Age, B - seniors aged 60 to 70 not attending classes at Universities of the Third Age, C - seniors aged 71 to 80 attending classes at Universities of the Third Age, D - seniors aged 71 to 80 not attending classes at Universities of the Third Age, E - seniors aged ≥81 years attending Universities of the Third Age classes, F - seniors aged ≥81 years not attending classes at Universities of the Third Age, H - value of the Kruskall-Wallis ANOVA test, p - level of statistical significance, NS – non significant. Red font in the supplementary tables indicates statistical significance.

**Explanation of abbreviations:** P-UTA”-” – seniors with primary education who do not attend classes at Universities of the Third Age, Z- UTA”+” – seniors with vocational education attending classes at Universities of the Third Age, Z- UTA”-” – seniors with education professional who do not attend classes at Universities of the Third Age, S- UTA "+" - seniors with secondary education attending classes at Universities of the Third Age, S- UTA "-" - seniors with secondary education not attending classes at Universities of the Third Age, W- UTA "+" – seniors with higher education attending classes at Universities of the Third Age, W- UTA”-” – seniors with higher education not attending classes at Universities of the Third Age, H – value of the Kruskall-Wallis ANOVA test, p – level of statistical significance

**Fig. SF6.** Characteristics of the study group, including the number of points obtained in the PAQE questionnaire, attendance at Universities of the Third Age and education

**Tab. ST11.** The value of statistical significance for multiple comparisons of the sum of points obtained in the PAQE physical activity questionnaire, taking into account level of education and participation in classes at Universities of the Third Age

| **p-value for multiple comparisons**  **Kruskal-Wallis test: H(6, N= 631)=38.84, p<0.001** | | | | | | | |
| --- | --- | --- | --- | --- | --- | --- | --- |
| **Sum of points obtained in the PAQE physical activity assessment questionnaire** | **P-UTW”-”** | **Z-UTW”+”** | **Z-UTW”-”** | **S-UTW”+”** | **S-UTW”-”** | **W-UTW”+”** | **W-UTW”-”** |
| **P-UTW”-”** | - | NS | NS | **0.029** | **0.007** | **<0.001** | **0.031** |
| **Z-UTW”+”** | NS | - | NS | NS | NS | **0.012** | NS |
| **Z-UTW”-”** | NS | NS | - | NS | NS | **<0.001** | NS |
| **S-UTW”+”** | **0.029** | NS | NS | - | NS | NS | NS |
| **S-UTW”-”** | **0.007** | NS | NS | NS | - | NS | NS |
| **W-UTW”+”** | **<0.001** | **0.012** | **<0.001** | NS | NS | - | NS |
| **W-UTW”-”** | **0.031** | NS | NS | NS | NS | NS | - |

**Wyjaśnienie skrótów:** P-UTA”-” – seniors with primary education who do not attend classes at Universities of the Third Age, Z- UTA”+” – seniors with vocational education attending classes at Universities of the Third Age, Z- UTA”-” – seniors with education professional who do not attend classes at Universities of the Third Age, S- UTA "+" - seniors with secondary education attending classes at Universities of the Third Age, S- UTA "-" - seniors with secondary education not attending classes at Universities of the Third Age, W- UTA "+" – seniors with higher education attending classes at Universities of the Third Age, W- UTA”-” – seniors with higher education not attending classes at Universities of the Third Age, H – value of the Kruskall-Wallis ANOVA test,
p – level of statistical significance, NS – no statistical significance. Red font in the supplementary tables indicates statistical significance.

**Explanation of abbreviations:** H - value of the Kruskall-Wallis ANOVA test, p - level of statistical significance, 1 - seniors living in the city, attending classes at Universities of the Third Age, 2 - seniors living in the city, not attending classes at Universities of the Third Age, 3 - seniors living in the countryside, attending classes at Universities of the Third Age, 4 - seniors living in the countryside, not attending classes at Universities of the Third Age

**Fig. SF7.** Characteristics of the study group, including the number of points obtained in the PAQE questionnaire, attendance at Universities of the Third Age and place of residence.

**Tab. ST12.** The value of statistical significance for multiple comparisons of the sum of points obtained in the PAQE physical activity assessment questionnaire, taking into account place of residence and participation in classes at Universities of the Third Age

| **p-value for multiple comparisons**  **Kruskal-Wallis test: H(3,N=631)=7.72, p=0.05** | | | | |
| --- | --- | --- | --- | --- |
| **Sum of points obtained in the PAQE physical activity assessment questionnaire** | **1** | **2** | **3** | **4** |
| **1** | - | NS | NS | NS |
| **2** | NS | - | NS | NS |
| **3** | NS | NS | - | NS |
| **4** | NS | NS | NS | - |

**Explanation of abbreviations:** H - value of the Kruskall-Wallis ANOVA test, p - level of statistical significance, 1 - seniors living in the city, attending classes at Universities of the Third Age, 2 - seniors living in the city, not attending classes at Universities of the Third Age, 3 - seniors living in the countryside, attending classes at Universities of the Third Age, 4 - seniors living in the countryside, not attending classes at Universities of the Third Age, NS - no statistical significance
